# Mindfulness Awareness Is Associated With a Lower Risk of Anxiety and Depressive Symptoms in Older Adults With Neurocognitive Disorders

**DOI:** 10.3389/fpsyt.2021.721583

**Published:** 2021-10-21

**Authors:** Linda Chiu Wa Lam, Allen T. C. Lee, Sheung Tak Cheng, Benjamin H. K. Yip, Wai Chi Chan, Ada W. T. Fung, Suk Ling Ma, Calvin P. W. Cheng, Ryan Kong, Henry T. S. Chiu, Frank H. Y. Lai, Samuel Yeung Shan Wong

**Affiliations:** ^1^Department of Psychiatry, Faculty of Medicine, The Chinese University of Hong Kong, Hong Kong, SAR China; ^2^Department of Health and Physical Education, The Education University of Hong Kong, Hong Kong, SAR China; ^3^The Jockey Club School of Public Health and Primary Care, The Chinese University of Hong Kong, Hong Kong, SAR China; ^4^Department of Psychiatry, University of Hong Kong, Hong Kong, SAR China; ^5^Department of Applied Social Sciences, Faculty of Health and Social Sciences, Hong Kong Polytechnic University, Hong Kong, SAR China; ^6^Department of Rehabilitation Sciences, Faculty of Health and Social Sciences, Hong Kong Polytechnic University, Hong Kong, SAR China

**Keywords:** neurocognitive disorder, dementia, depression, anxiety, dispositional mindfulness

## Abstract

**Background:** Apart from depressive disorders, there are great interests in adopting mindfulness based interventions (MBIs) for other mental health conditions. Depression and anxiety are common in people with neurocognitive disorders (NCD). The potential of MBIs as an adjuvant treatment in this cognitively at-risk group should be further explored.

**Objectives:** The current study explored the association between depression and anxiety symptoms with dispositional mindfulness in older adults, and if same association stays in the context of cognitive impairment.

**Methods:** The Hong Kong Mental Morbidity Survey for Older People (MMSOP) is an ongoing epidemiology study of the prevalence of neurocognitive and mental disorders in adults aged 60 years or over in Hong Kong. MMSOP evaluated cognitive function, psychiatric symptoms (Clinical Interview Schedule-revised, CIS-R), chronic physical disease burden, psychosocial support, and resilience factors, including dispositional mindfulness as measured by the Mindful Attention Awareness Scale (MAAS). We analyzed the impact of MAAS on CIS-R and potential moderation effects of mindfulness.

**Results:** In March 2021, 1,218 community dwelling participants completed assessments. The mean age of the sample is 69.0 (SD 6.9) years. Eight hundred and two participants (65.7%) were not demented (CDR 0) and 391 (32%) and 25 (2%) were categorized as having mild NCD (CDR 0.5) and major NCD (CDR 1 or more), respectively. One hundred forty-three (11.7%) satisfied ICD-10 criteria for anxiety or depressive disorder as measured by CIS-R. Linear regression analysis showed that female gender, CIRS, and MAAS scores were significant factors associated with CIS-R scores. MAAS scores moderated and attenuated the impact CIRS on CIS-R (adjusted *R*^2^ = 0.447, *p* < 0.001). MAAS scores remained as significant moderator for CIRS in patients with NCD (CDR ≥ 0.5) (adjusted *R*^2^ = 0.33, *p* < 0.001).

**Conclusion:** Interim findings of the MMSOP suggested that dispositional mindfulness is associated with lower level of mood symptoms in community dwelling older adults in Hong Kong. The interaction effects further suggested that high mindful awareness may reduce the adverse effects of chronic physical morbidity on mental health. The observation stayed in the participants with cognitive impairment. We should further explore MBIs as a non-pharmacological treatment for in older adults at-risk of physical morbidity and cognitive decline.

## Introduction

Mindfulness has been a cherished spiritual practice aiming to achieve a state of serenity in different religions over the world. The tradition of mindfulness practice, while described in different terms, could be tracked back thousands of years. Some key concepts of dispositional (also referred to as trait) mindfulness describes a personal attribute in moment-to-moment conscious awareness of internal and external sensation, own emotional state with self-regulation of affective response with a non-judgemental and neutral attitude (1). In the past few decades, considering that there may be changes in the ability to become more mindful, mindfulness related practices such as meditation have been turned into an emerging strategy for the management of mental and physical health problems.

Mindfulness based interventions (MBIs), based on mindfulness approach, are enriched with conventional psychological treatment concepts. MBIs are well-recognized for its benefits on relapse prevention of recurrent depressive disorders (2). In a meta-analysis of 1,258 patients with a 60-week follow up period, those who received mindfulness based cognitive therapy (MBCT) had a reduced risk for relapse of depression (hazard ratio, 0.69, 95% CI, 0.58–0.82). There was also a significant interaction between baseline depression scores and MBCT treatment effects. A higher baseline depression was associated with a larger treatment effect (hazard ratio, 0.80, 95% CI, 0.66–0.97) (3). Apart from mental disorders, the findings of meta-analyses generally supported positive benefits of MBIs in pain and cancer-associated mood syndromes, although the effect sizes and sustainability of benefits are less consistent compared to recurrent depression (4, 5). In a 12 week randomized controlled trial of mindfulness vs. waitlist control, MBI demonstrated large effect size (*d* = 0.85) on interoceptive awareness and self-regulation in war veterans with stress-related disorders (6).

The therapeutic mechanisms by which MBIs brought about better mood and mental health may be diverse. In a review paper discussing the relationship between mindfulness meditation and psychopathology, the components of mindfulness practice in the promoting self-regulation of affect and cognition are examined. The review offered support for MBIs as an intervention for different mental disorders when mood and affective regulations are frequently disrupted (7). Some studies suggested that older adults who assumed mindfulness practice regularly are associated with higher cognitive function and less age-related brain degenerative responses, with the prefrontal, cingulate, and insular cortex implicated as significant regions of interests (8–10). It poses a challenging question—Will MBI work only in people with high cognitive skills? Apart from progressive cognitive and functional deterioration, depressive and anxiety symptoms are not only prevalent in people with neurocognitive disorders (NCD), they also jeopardize life quality and poses risks to patients and carers. However, meta-analysis studies on antidepressant treatment in people with dementia and behavioral disturbances showed variable treatment effects (11, 12). Adjuvant non-drug measures, including lifestyle modifications, may have a role for optimization of mood symptoms. In a recent review paper, the authors examined MBIs and cognitive training in people with mild NCD. The results suggested preliminary evidence for positive effects on cognition and mood in this cognitively at-risk group (13).

Before embarking on the development of MBIs for mood management in people with NCD, we explored the association between dispositional mindfulness and mood symptoms in a randomly recruited community sample of older adults in Hong Kong. We aimed to address two questions in this report. First, would dispositional mindfulness be associated with fewer mood symptoms? Second, would the association between mindfulness and mood symptoms differ between cognitively healthy older adults and those with NCD?

## Methods

### Participants and Study Design

Subjects recruited in the present report were community dwelling older participants of the ongoing Hong Kong Mental Morbidity Survey for Older People (HKMMSOP). MMSOP is a cross-sectional epidemiological study funded by the Food and Health Bureau of the Hong Kong SAR. The main study aims to evaluate the prevalence of NCDs and mental disorders in older adults in Hong Kong. Data collection started in 2019, and the target sample size will be 6,000. Potential participants were identified through postal invitations from random sampling of regional addresses quarters provided by the Census and Statistics Department. Selected households with adults at or above 60 years old interested to participate in the study contacted our research office for assessments. For each consented participant, we conducted cognitive, mental and physical health, as well as lifestyle enquiries. The current report represented the interim findings of the initial 1,500 participants of the HKMMSOP, from which 1,218 completed the questionnaire on dispositional mindfulness.

### Ethical Considerations

Institutional Review Board approvals have been obtained from the Survey and Behavioral Research Ethics Committee and Clinical Research Ethics Committee of the Chinese University of Hong Kong. Written informed consent was obtained for all participants of the current report. A brief report of the assessment results was prepared as his/her personal health record for each participant. For people with NCD or psychiatric disorders but not yet assessed under clinical settings, we prepared written referral letters for further care at appropriate settings. The study has been registered under China Clinical Registry (ChiCTR1900024349).

### Assessments

Apart from demographic profile, cognitive function, depression and anxiety symptoms, chronic physical morbidity, dispositional mindfulness, and sense of loneliness were assessed. Details of the assessment instruments are listed below.

### Basic Demographic Information

- Global Cognition was assessed by the Clinical Dementia Rating (CDR). CDR is a clinician rated assessment on six areas of cognition and everyday function, which includes memory, orientation, judgment and problem solving, community affairs, home and hobbies, and personal care (14). CDR determines overall cognition from normal (not-demented, CDR = 0) to severe dementia (CDR = 3). In the paper, we adopted a reference with CDR 0.5 for mild NCD and CDR ≥ 1 for major NCD.
- The locally validated Hong Kong Montreal Cognitive Assessment (MoCA) was administrated to determine a global cognitive function score in the current study (15). The MoCA is a Chinese validated version of MoCA, which examines cognitive domains including memory, attention, language, visuospatial skills, orientation, abstraction, executive function, and calculation (16). Studies in Hong Kong older population suggested that MoCA is sensitive in detecting executive function changes in subcortical vascular NCD, which is highly prevalent in Chinese population.
- Mood symptoms were evaluated with the Revised Clinical Interview Schedule (CIS-R) (17, 18). The CIS-R is a structured psychiatric assessment interview schedule tapping 14 groups of non-psychotic psychological symptoms in the week prior to interview. The symptom clusters and scores were integrated to form ICD-10 diagnoses of six groups of common mental disorders including generalized anxiety disorder (GAD), mixed anxiety and depressive disorder, depressive episode, phobias, obsessive compulsive disorder (OCD), and panic disorder. A cutoff score of 12 is indicative of syndromal diagnosis of depressive or anxiety disorders both in the Chinese and original English versions.
- Chronic disease burden was measured by the Geriatric version of the Cumulative Illness Disease Raring Scale (CIRS) (19). CIRS measures morbidity of 14 categories of physical and psychiatric diseases. A score of 1–4 for each system disease indicates severity by which a condition affects life and urgency of treatment. The CIRS-Geriatric version estimated a severity index to show over disease severity in geriatric population.
- Dispositional mindfulness was measured by the Mindful Attention Awareness Scale (MAAS) (1). The MAAS is a 15-item scale used to assess participants' attention awareness to present moment experience. MAAS has a score of 1–6, with higher score indicated higher degree for attention to present states. MAAS has demonstrated psychometric properties with a 1-factor structure; it was validated across different cultures and populations (20, 21). We asked about the practice duration of mindfulness activities with a questionnaire on leisure activities. Examples of mindfulness activities included writing reflective diaries, meditation, quiet sitting, reading religious texts, and making prayer.
- Subjective loneliness was measured by the Chinese 6-item De Jong Gierveld Loneliness Scale. The dimensions of social and emotional loneliness have been identified as important risk factor for poor mental health in the older population. The Chinese version has been validated with satisfactory psychometric properties (22).

### Statistical Analyses and Power Estimation

The weighted population prevalence of neurocognitive and mental disorders in older people will be computed when the field work of MMSOP has been completed. The current report is a preliminary analysis of the first 1,218 subjects. We focused on the associative factors of depressive and anxiety symptoms as measured by CIS-R, and dispositional mindfulness as a potential modulator between the risk factors and CIS-R scores is evaluated. We estimated the sample with linear regression model using G^*^Power3.1. For a small effect size of 0.02 and 7 predictors (age, gender, education, MAAS, MoCA, CIRS, loneliness) for CIS-R scores as dependent variable, 725 subjects are required to achieve a power of 0.8.

We examined the prevalence of depressive and anxiety disorders, and correlations between associative factors and the CIS-R scores. The variables examined included demographic factors, chronic physical morbidity (CIRS), global cognitive function (MoCA), dispositional mindfulness (MAAS scores), and loneliness scores. Variables having potentially significant associations with CIS-R scores are entered as independent variables in linear regression analysis. CIS-R scores were the dependent variable, with interaction effects between MAAS and risk factors (centered) computed in the regression analysis. To further explore if MAAS scores are associative factor for mood symptoms in cognitive compromised individuals, regression analyses were repeated in CDR 0 (cognitively healthy) and CDR ≥0.5 (NCD) groups, respectively. SPSS V24 was used to compute the statistical analyses, with significant level set at *p* < 0.05.

## Results

### Sample Characteristics

In this interim report, 1,218 participants have been assessed for cognitive function, psychiatric symptoms, and dispositional mindfulness. The mean age of the sample is 69.0 (SD 6.9) years; 555 (45.5%) were men, with an average education of 8.9 (SD 4.3) years. The average MoCA score was 24.7 (SD 3.9). As for global cognitive status, 802 (65.7%) was not demented (CDR 0). Three hundred ninety-one (32%) and 25 (2%) were categorized as having mild NCD (CDR 0.5) and major NCD (CDR 1 or more), respectively.

The average MAAS score is 5.3 (SD 0.6), indicating the range for scoring MAAS is relatively high in this subject sample (Table 1). Nine hundred and ninety participants (81%) had no regular mindfulness activities. For those who practice mindful activities regularly, the average year of practice was 23.4 (SD 18.6). There was no difference in MAAS scores between those who practiced (5.3, SD 0.6) and did not practice (5.3, SD 0.5) mindful activities (*t*-test, *p* = 0.46). We observed a positive association between years of mindfulness practice and MAAS scores (*r* = 0.17, *p* = 0.009) and education (*r* = 0.0.18, *p* = 0.006). Higher MAAS scores are associated with female gender (*t* = 4.99, *p* < 0.001), older age (*r* = 0.096, *p* = 0.001), and lower CIRS (*r* = −0.187, *p* < 0.001). In regression analysis of demographic factors associated with MAAS scores, only duration of practice was significant (*p* = 0.02), while age, gender, and years of education were not significant (linear regression, *R*^2^ = 0.03).

**Table 1:** Demographic characteristics of sample.

	**Total**	**CDR 0**	**CDR 0.5**	**CDR ≥ 1**	***P-value* ^**b**^**
	**(*n =* 1218)**	**(*n =* 802)**	**(*n =* 391)**	**(*n =* 25)**	
Age (years)	69.0 ± 6.9	67.3 ± 6.0	71.9 ± 7.4	76.5± 7.6	<0.001
Gender (*n*, %)^a^					
Male	555 (45.5%)	367 (45.8%)	180 (46.0%)	8 (32.0%)	0.387
Female	663 (54.4%)	435 (54.2%)	211 (54.0%)	17 (68.0%)	
Education (years)	8.9 ± 4.3	9.7 ± 4.1	7.4 ± 4.2	5.5 ± 4.0	<0.001
MoCA	24.7 ± 3.9	26.3 ± 2.6	22.0 ± 3.8	15.2 ± 5.3	<0.001
CIS-R	5.0 ± 8.6	4.2 ± 7.8	6.2 ± 7.2	10.4 ± 21.0	<0.001
CIRS	4.1 ± 2.7	3.7 ± 2.2	5.0 ± 3.1	5.6 ± 3.5	<0.001
MAAS	5.3 ± 0.6	5.4 ± 0.5	5.2 ± 0.6	5.0 ± 1.0	<0.001
Loneliness	2.8 ± 1.3	2.7 ± 1.3	2.8 ± 1.3	2.7 ± 0.9	0.335

*MoCA, Montreal Cognitive Assessment; CIS-R, Clinical Interview Schedule – revised; CIRS, Cumulative Illness Disease Raring Scale; MAAS, The Mindful Attention Awareness Scale; Loneliness, 6-item De Jong Gierveld Loneliness Scale; CDR, Clinical Dementia Rating.*

a *Chi Square.*

b *ANOVA, analysis of variance*.

### Prevalence of Mood Symptoms and Association With Dispositional Mindfulness

The average CIS-R scores was 5.0 (SD 8.6). One hundred forty-three (11.7%) satisfied ICD-10 criteria for one anxiety or depressive disorder. Of them, 67 (5.5%) had generalized anxiety disorder (GAD), 30 (2.5%) had depressive disorder, and 52 (4.2%) had mixed anxiety and depressive disorder, with a proportion suffering from comorbid mood disorders. The CIS-R scores were associated with female gender (*t* = 4.9, *p* < 0.001), lower educational attainment (*r* = −0.07, *p* = 0.013), lower MoCA score (*r* = −0.11, *p* < 0.001), higher CIRS score (*r* = 0.23, *p* < 0.001), higher loneliness (*r* = 0.07, *p* = 0.015), and lower MAAS score (*r* = −0.35, *p* < 0.001). Age is not significantly associated with CIS-R scores (Table 2).

**Table 2:** Clinical correlates of CIS-R scores.

	**Correlations (*r*)^**a**^**	***P-value***
Gender^b^	4.88^***^	<0.001
Age (years)	0.002	0.954
Education (years)	−0.07^*^	0.013
MoCA	−0.11^***^	<0.001
MAAS	−0.35^***^	<0.001
CIRS	0.23^***^	<0.001
Loneliness	0.07^*^	0.015

*MoCA, Montreal Cognitive Assessment; CIS-R, Clinical Interview Schedule – revised; CIRS, Cumulative Illness Disease Raring Scale; MAAS, The Mindful Attention Awareness Scale; Loneliness, 6-item De Jong Gierveld Loneliness Scale.*

* *Significant at p < 0.05.*

*** *Significant at p < 0.001.*

a *Pearson correlations.*

b *t-test*.

### Mood Symptoms and Dispositional Mindfulness

**Figure 1:**
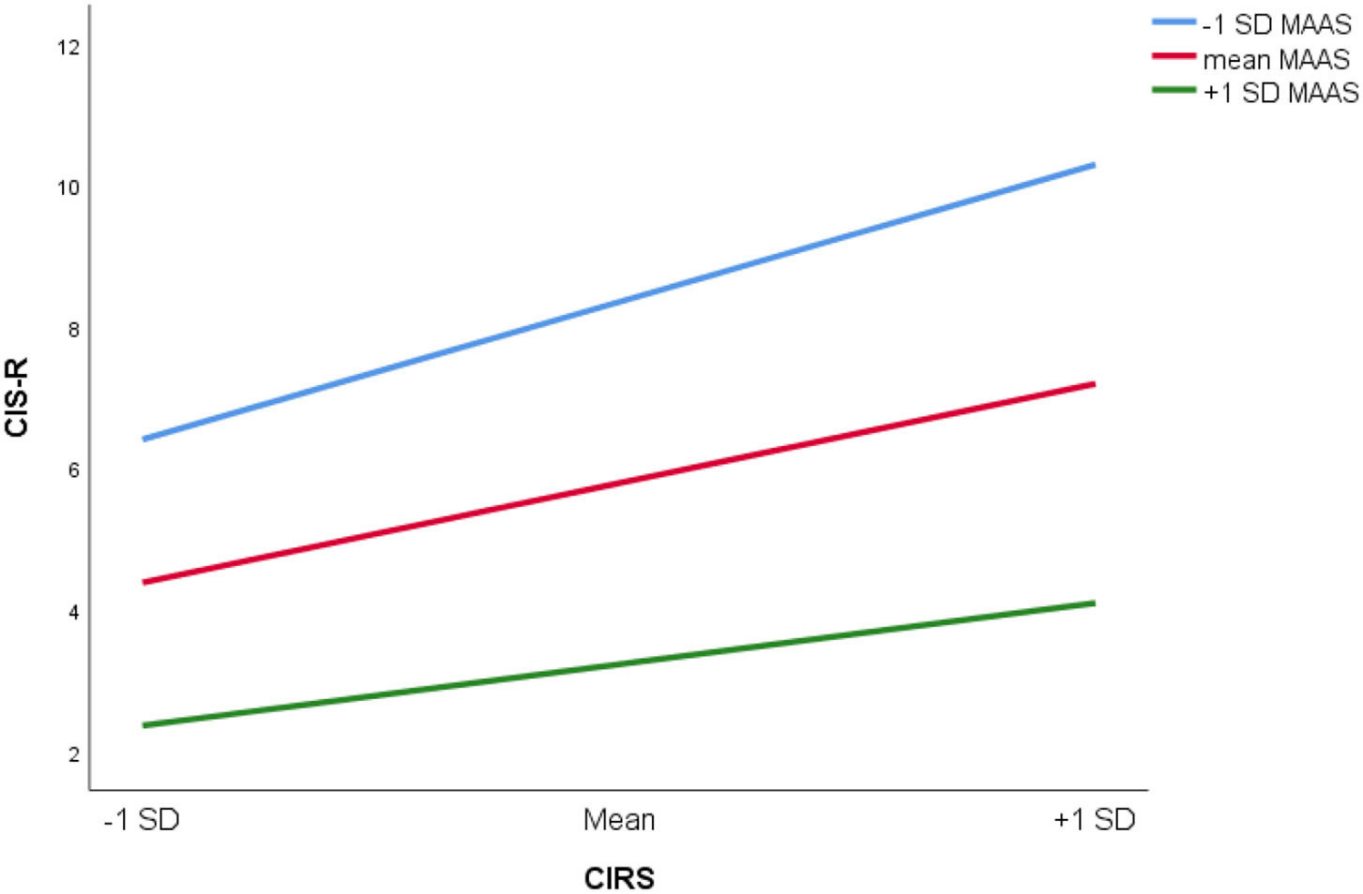
Relationship between CIS-R (Revised Clinical Interview Scale) and CIRS (Cumulative Illness Rating Scale) at MAAS (Mindful Attention Awareness Scale) equal to −1 SD, mean and +1 SD.

Female gender, higher CIRS, and lower MAAS scores are significant factors for higher CIS-R scores (linear regression, adjusted *R*^2^ = 0.439, *p* < 0.001). MAAS scores interacted with CIRS on CIS-R scores. Higher level of dispositional mindfulness attenuated the association between CIRS and CIS-R scores (Δ*R*^2^ = 0.007, *p* = 0.001) (Table 3). The regression analysis was repeated in patients with CDR = 0 and CDR ≥ 0.5 (NCD group), respectively. For CDR 0 group, female gender, lower MAAS, and higher CIRS scores were associated with higher CIS-R (adjusted *R*^2^ = 0.348, *p* < 0.001). Interaction effect between MAAS and CIRS was not significant in this group (Δ*R*^2^ = 0.004, *p* = 0.07). For CDR ≥ 0.5 group, female gender, higher CIRS, and lower MAAS scores were associated with higher CIS-R scores (adjusted *R*^2^ = 0.320, *p* < 0.001). Interaction effects between MAAS and CIRS (interaction effect, Δ*R*^2^ = 0.011, *p* = 0.013) groups are shown (Table 4, Figure 1).

**Table 3:** Factors associated with CIS-R scores (All participants).

	**Model 1** ^**a**^			**Model 2** ^**b**^		
**Factors**	***Standardized***	***t***	***P***	***Standardized***	***t***	***P***
	**β**			**β**		
MAAS	−0.34^***^	−12.43	<0.001	−0.20^***^	−4.13	<0.001
Female	0.09^***^	3.38	0.001	0.09^**^	3.43	0.001
Education (years)	0.006	0.18	0.854	0.003	0.08	0.934
CIRS	0.19^***^	7.10	<0.001	0.87^***^	4.17	<0.001
MoCA	−0.04	−1.21	0.228	−0.04	−1.23	0.220
Loneliness	0.01	0.55	0.583	0.01	0.32	0.749
MAAS^*^CIRS				−0.68^**^	−3.28	0.001

*CIS-R, Clinical Interview Schedule – Revised; CIRS, Cumulative Illness Disease Raring Scale; MAAS, The Mindful Attention Awareness Scale; MoCA, Montreal Cognitive Assessment; MP Duration, years of mindfulness practice.*

* *Significant at p < 0.05.*

** *Significant at p < 0.01.*

*** *Significant at p < 0.001.*

a *Linear regression adjusted R^2^ = 0.439, p < 0.001.*

b *Linear regression adjusted R^2^ =0.447, ΔR^2^ = 0.007 (p = 0.001)*.

**Table 4A:** Factors associated with CIS-R scores (Stratified with CDR status).

	**Model 1** ^**a**^			**Model 2** ^**b**^		
**Factors**	***Standardized β***	***t***	***P***	***Standardized β***	***t***	***P***
MAAS	−0.27^***^	−7.74	<0.001	−0.16^*^	−2.47	0.014
Female	0.08^*^	2.33	0.020	0.08^*^	2.41	0.016
Education	−0.02	−0.45	0.651	−0.02	−0.47	0.64
CIRS	0.15^***^	4.37	<0.001	0.72^*^	2.25	0.025
Loneliness	−0.002	−0.05	0.962	−0.006	−0.18	0.859
MAAS^*^CIRS				−0.56	−1.79	0.07

*CDR = 0.*

*CIS-R, Clinical Interview Schedule – Revised; CIRS, Cumulative Illness Disease Raring Scale; MAAS, The Mindful Attention Awareness Scale; MoCA, Montreal Cognitive Assessment.*

* *Significant at p < 0.05.*

*** *Significant at p < 0.001.*

a *Linear regression adjusted R^2^ = 0.348, p < 0.001.*

b *Linear regression adjusted R^2^ = 0.353, ΔR^2^ = 0.004, (p = 0.07)*.

**Table 4B:** CDR ≥ 0.5.

	**Model 1** ^**a**^			**Model 2** ^**b**^		
**Factors**	***Standardized β***	***t***	***P***	***Standardized β***	***t***	***P***
MAAS	−0.44^***^	−10.37	<0.001	−0.28^***^	−3.58	<0.001
Female	0.13^**^	3.05	0.002	0.13^**^	2.97	0.003
Education	0.03^*^	0.69	0.490	−0.03	0.56	0.578
CIRS	0.25^***^	5.93	<0.001	0.97^**^	3.33	0.001
Loneliness	0.05	1.15	0.252	0.04	0.94	0.346
MAAS^*^CIRS				−0.73^*^	−2.50	0.013

*CIS-R, Clinical Interview Schedule – Revised; CIRS, Cumulative Illness Disease Raring Scale; MAAS, The Mindful Attention Awareness Scale; MoCA, Montreal Cognitive Assessment.*

* *Significant at p < 0.05.*

** *Significant at p < 0.01.*

*** *Significant at p < 0.001.*

a *Linear regression adjusted R^2^ = 0.320, p < 0.001.*

b *Linear regression adjusted R^2^ = 0.330, ΔR^2^ = 0.011 (p = 0.013)*.

## Discussion

Our findings suggested about one in nine community dwelling older adults suffered from anxiety and/or depressive disorders in Hong Kong. This approximated previous local studies in late life mood disorders and suggested that participants of this preliminary analysis are representative of the older population of Hong Kong (23). It is not unexpected that female gender, poorer physical health, and lower cognitive function are associated with high risks of depression and anxiety. While these unfavorable physical and psychosocial risks are well-recognized, many of these risks are enduring in later life and difficult to modify. Pharmacological interventions remain as important treatment options. However, judicious use of drugs has been limited by comorbid medical conditions and sensitivity to adverse effects of medication. The challenges on drug use are of particular concern in people suffering from NCD.

Dispositional mindfulness is examined as potentially modifiable factor for mental health in our report. We found that MAAS scores are associated with lower level of mood symptoms, as reflected by lower CISR scores. As dispositional mindfulness possibly represented a “negative” risk factor for mood disorders, we tried to explore if dispositional mindfulness act through modulating the association between physical risk factors and mood symptoms. Our findings suggested that MAAS scores interacted with chronic medical disease burden on CIS-R scores. When a person is more “mindful,” the association between physical symptoms and negative mood states appeared to be attenuated. MAAS captures a person's subjective attention awareness of daily experiences. It may impact on mental health through different pathways, from enhanced awareness of body sensation and possibly discomfort, to better emotional control. The better awareness may also help the individual to derive strategies in adapting physical limitations caused by chronic medical illness.

We further stratified the association and modulating effects of MAAS in different cognitive groups. MAAS and CIRS stayed as risk and “protective” factors in both cognitively healthy and impaired groups. The influence of chronic medical morbidity remained a significant risk factor for depression and anxiety, while a higher level of MAAS decreased the negative impact of poor physical state on mood symptoms, as well as being an independent factor on mood symptoms. The findings gave some inference that dispositional mindfulness is associated with lower mood symptoms, part of which may act through moderating the distress associated with physical morbidity.

Not until recently, MBIs have emerged as a potential option for the management of depression and anxiety in older adults. A randomized controlled trial of mindful yoga on 138 Chinese patients with Parkinson's disease indicated that yoga intervention was superior to stretching and resistance training in different mental health outcomes (24). Compared with cognitive behavioral therapy, which emphasizes on rectification of interpretation biases and dysfunctional beliefs, MBIs focus on attention awareness and non-judgmental perception of internal sensory experiences. The theoretical constructs of MBIs could be more applicable to older adults who are more likely to be living with chronic health limitations and unresolvable psychosocial adversities. The current study offered some support to the development of MBIs in people with compromised physical and cognitive function. Medical morbidity is highly prevalent in the older population, and chronic body symptoms are highly distressing. Attentional awareness to everyday experience helped to reduce preoccupation with negative affect associated with physical discomfort. It may also lead better attention to physical conditions and adherence to medical treatment.

This study should be interpreted in the context of its limitations. First, this is a preliminary report of an ongoing epidemiological survey. Cross-sectional single assessment only offers information on association and did not give any directional or causative information. Second, we only used MAAS score to evaluate the level of attention awareness. We did not find differences in MAAS scores between participants who practiced and did not practice mindful activities. This may be related to a wide range of duration of practice. On the other hand, we did find a positive association between MAAS scores and year of practice. This may suggest that an attitude of mindfulness is potentially adaptable as a long term lifestyle habit to improve mindful awareness. We hoped that prospective randomized controlled trials (RCT) on MBIs in people with mild NCD would give empirical evidence on its efficacy on mental health. Third, the sample size for major NCD is limited. This may limit the interpretation for the group of subjects with clinical dementia. In a RCT investigating the effect of mindfulness and cognitive stimulation in subjects with mild to moderate Alzheimer's disease, the findings suggested that both mindfulness and cognitive stimulation showed less deterioration in cognitive function at 2 years (25). This added support to the development of MBIs in people with NCDs. Our findings further suggested that, apart from cognitive abilities, mental health parameters should also be considered as important outcome measure in future clinical trials.

In older adults experiencing cognitive decline, MBIs would serve as a cognitive stimulation paradigm. Attention and executive function training embedded in the mindful practice may help people with cognitive impairment to assume more adaptive functional skills. Mindfulness practice may also improve mood regulation and mental health outcomes. As suggested by a recent review, MBIs have a potential for wider utilization as health promotion strategies in people with cognitive impairment, but better design clinical trials are required to address methodological limitations that affect generalizability and clinical applications (26).

## Data Availability Statement

The raw data supporting the conclusions of this article will be made available by the authors, without undue reservation.

## Ethics Statement

The studies involving human participants were reviewed and approved by Survey and Behavioral Research Ethics Committee and Clinical Research Ethics Committee of the Chinese University of Hong Kong. The patients/participants provided their written informed consent to participate in this study.

## Author Contributions

LL is responsible for conceptualization of study, supervision of study logistics, data analysis, and preparation of manuscript. AL, WC, CC, AF, FL, and SM are responsible for conceptualization of study, training and supervision of assessment, and preparation of manuscript. RK and HC are responsible for data collection, statistical analysis and preparation of manuscript. SC, BY, and SW are responsible for conceptualizing the study, data analysis, and preparation of manuscript. All authors contributed to the article and approved the submitted version.

## Funding

The Hong Kong Mental Morbidity Survey for Older People is funded by the Health and Medical Research Fund of the Food and Health Bureau of the government of Hong Kong SAR [Reference: MHS-P1(Part 3)].

## Conflict of Interest

The authors declare that the research was conducted in the absence of any commercial or financial relationships that could be construed as a potential conflict of interest.

## Publisher's Note

All claims expressed in this article are solely those of the authors and do not necessarily represent those of their affiliated organizations, or those of the publisher, the editors and the reviewers. Any product that may be evaluated in this article, or claim that may be made by its manufacturer, is not guaranteed or endorsed by the publisher.
